# Comparing P53 expression and genome-wide transcriptome profiling to Comet assay in lymphocytes from melanoma patients and healthy controls

**DOI:** 10.1038/s41598-023-44965-z

**Published:** 2023-11-01

**Authors:** Mojgan Najafzadeh, Parisa Naeem, Nader Ghaderi, Shohreh Jafarinejad, Zahra Karimi, Mehran Ghaderi, Pouria Akhbari, Rojan Ghaderi, Pedram Farsi, Andrew Wright, Diana Anderson

**Affiliations:** 1 School of Life Sciences, University of Bradford, Richmond Road, Bradford, BD7 1DP West Yorkshire UK; 2 Bradford Teaching Hospitals NHS Foundation Trust, St Luke’s Hospital, Little Horton Lane, BD5 0NA UK; 3 Division of Pathology F46, Department of Laboratory Medicine, Karolinska Institute, Karolinska University Hospital, Huddinge, 141 86 Stockholm, Sweden; 4 Institute of Biomedical and Clinical Science, College of Medicine and Health, University of Exeter, Exeter, EX2 5DW UK; 5 Department of Medicine, Imperial College London, London, SW7 2BX UK; 6 Department of Clinical Pathology and Cytology, Karolinska University Hospital, 141 86 Stockholm, Sweden

**Keywords:** Cancer, Cell biology, Genetics, Biomarkers

## Abstract

This study compared the expression of TP53 in lymphocytes from malignant melanoma (MM) patients with positive sentinel nodes to healthy controls (HCs) following exposure to various doses of UVA radiation. The Lymphocyte Genome Sensitivity (LGS) assay indicated significant differences in DNA damage in lymphocytes between MM patients and HCs. qPCR data demonstrated an overall 3.4-fold increase in TP53 expression in lymphocytes from MM patients compared to healthy controls, following treatment with 0.5 mW/cm^2^ UVA radiation. Western blotting confirmed that p53 expression was increased in MM lymphocytes following UVA exposure compared to healthy individuals. Genome transcriptome profiling data displayed differences in gene expression between UVA-treated lymphocytes from MM patients and HCs. Peripheral lymphocytes from MM patients are more susceptible to the genotoxic effects of UVA compared to healthy individuals. Our previous studies showed that UVA exposure of various intensities caused significant differences in the levels of DNA damage between lymphocytes from cancer patients compared to HCs through the LGS assay. The present study’s results provide further credibility to the LGS assay as a screening test for cancer detection. Peripheral lymphocytes could be a promising blood biopsy biomarker for staging of carcinomas and prevention of carcinoma progression at early stages.

## Introduction

In most countries, a sentinel lymph node biopsy is considered an important staging procedure for melanoma^1,2^. Cutaneous melanoma accounts for 55,500 deaths globally. Once melanoma metastasises, it becomes life-threatening, and its incidence rate and mortality depend upon early detection criteria. In cancer patients, positive sentinel nodes indicate metastasis and is believed to be an important prognostic factor. Screening sentinel nodes has shown promising results in distinguishing slow-progressing cancers from metastasised tumours^3^.

Established risk factors for malignant melanoma include ultraviolet radiation (UVA and UVB) from tanning beds, direct sun exposure, sunburns, dysplastic naevi, family history of melanoma, freckles, light hair and light skin^4^ In primary and malignant melanoma, p53 tumour-suppressing protein is expressed at high levels^5,6^ It has been suggested that p53 is not mutated in melanoma. However, the abrogation of its pathway may result from the upregulation of MDM2 or downregulation of p14ARF and p16INK4a, all of which are members of the TP53 family. The primary function of p53 is to inhibit the accumulation of free radicals in the cells by stimulating a series of events such as apoptosis, cell cycle arrest and senescence^7^ Prolonged exposure to UVA increases the production of free radicals in skin cells, leading to photocarcinogenesis. Unlike UVA, UVB requires high doses to promote skin cancer development. Skin cancer originating from UVA is believed to originate from DNA damage that results in the accumulation of reactive oxygen species (ROS)^8^

Lymphocytes play a role in all three stages (early, intermediate and late) of melanoma regression^9^ Lymphocytes are tumour-infiltrating and have a significant role in melanoma prognosis^10^ Additionally, tumour cells secrete inflammatory chemokines which impact lymphocyte function and induce oxidative stress. At least 48 chemokines (in 4 groupings, i.e. CC, CXC, C, and CX3C) and more than 20 chemokine receptors were detected in the various types of response to stimuli that result in inflammation^11,12^

Malignant transformation of lymphocyte cells in the presence of chronic inflammation (involving cytokines and chemokines such as TNFα, IL1, IL6, IL8, CXCL12 and CXCR4) drives cells to reform DNA, undergo angiogenesis, and undergo the inactivation of suppressor genes^13,14^

Transcriptome sequencing is a sequencing technology used to determine the level of RNA expression in tissues. Sequence data can be aligned to a reference genome to build full-length transcripts. Transcriptome sequencing data can be used to define novel transcripts, leading to the discovery of neoantigens^15,16^

In this assay, transcriptional changes were assessed in a limited number of genes during tissue culture (ex-vivo). Changes were mainly found in genes involved in molecular functions which affect the stability or regulation of cellular compartments. Consistent with histomorphological analysis, we observed an upregulation of pathways related to apoptosis in a cultured tumour with decreased tissue viability, while they were not upregulated in the tumour cultures with minimal tissue loss^17^

The haematopoietic system is at constant risk of oxidative stress due to exogenous and endogenous mutagens which increase the risk of carcinoma. Different biomarkers exist to identify different types of carcinomas, including imaging, biopsies and blood tests with high specificity in mutation detection^18^ In malignant melanoma, efficient and affordable staging techniques are not readily available. However, Anderson et al. demonstrated the use of the Lymphocyte Genome Sensitivity (LGS) assay wherein peripheral blood lymphocytes can be used as biomarkers in the detection of different types of tumours. At the time of the study, the authors examined blood samples from 208 individuals, but to this date, 900 samples have been analysed, all showing the same patterns of response and 90+ % predictivity for cancer. Our previous studies demonstrated that lymphocytes from cancer patients show a greater level of DNA damage than lymphocytes from healthy individuals^19,20^

**Figure 1:**
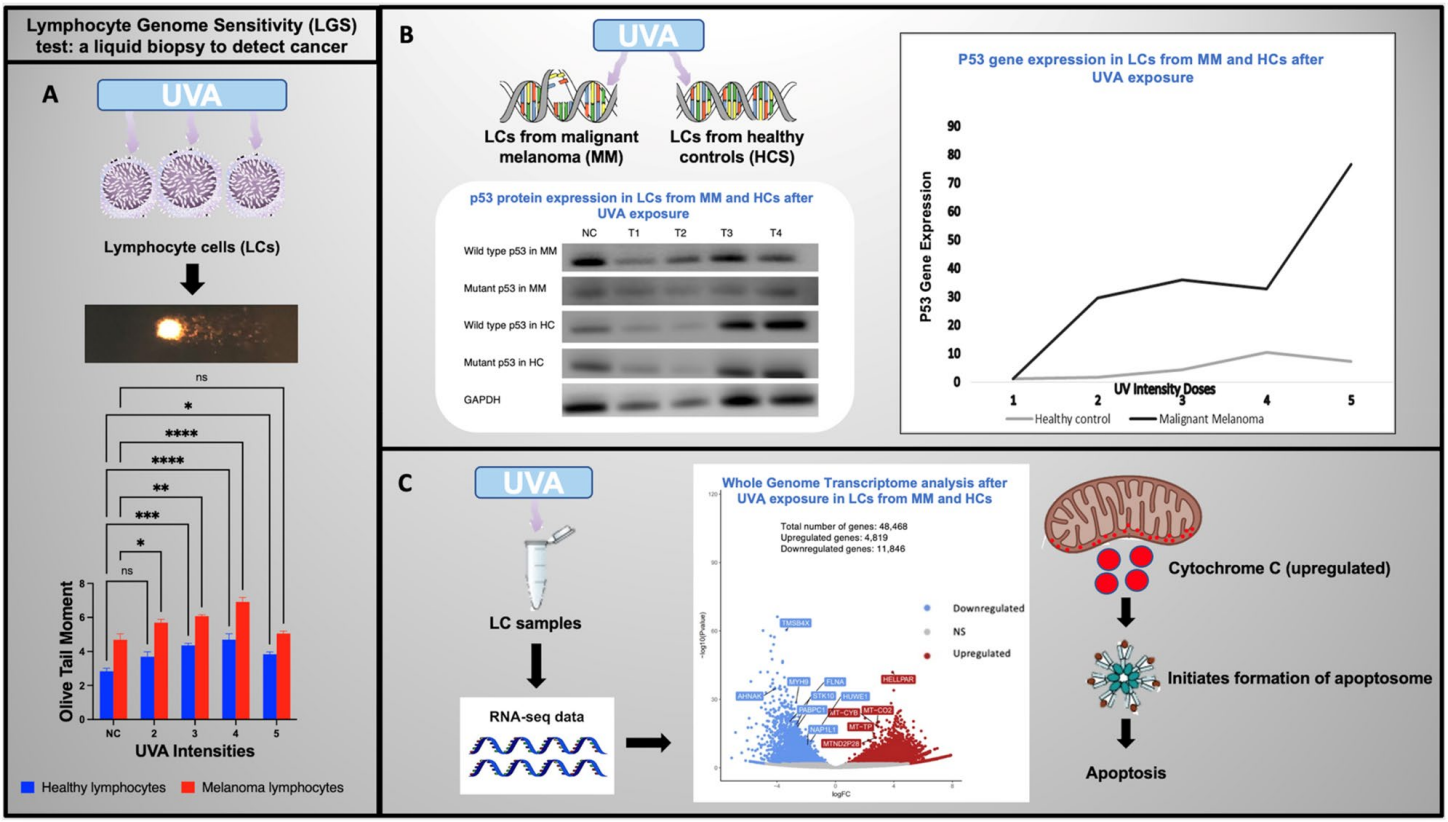
A summary of the objectives and results of this study.

In the present study, we investigated the in-vitro responses of peripheral blood lymphocytes exposed to UVA. These lymphocytes were obtained from melanoma patients at precancerous and malignant stages possessing positive sentinel nodes. The LGS assay, quantitative PCR (qPCR) and Western blotting were used to evaluate DNA genomic integrity and TP53 and p53 expression following exposure to UVA radiation (Fig. 1).

## Materials and methods

### Blood samples

Peripheral blood samples were obtained via venepuncture from malignant melanoma (MM) patients during their initial visit to the fast-track clinic or in the event of a recurrence of melanoma. In the same manner, peripheral blood samples were also procured from healthy controls (HCs). All samples were obtained when the patients were not undergoing any treatments. Prior to the sample collection, informed consent was obtained. Ethical approval for the collection of blood samples was granted by the University of Bradford Sub-Committee for Ethics in Research involving Human Subjects (Ref: 0405/8) and the Research Support and Governance office, Bradford Teaching Hospitals, NHS Foundation (Ref: RE DA 1202).

It is necessary to confirm that all methods were conducted in compliance with the appropriate guidelines and regulations. This can be achieved by stating such information in the “Methods” section.

### The LGS assay

Lymphocytes were isolated from the blood of patients and healthy volunteers using Lymphoprep (Axis Shield, Norway). Lymphocytes were embedded on slides pre-coated with 0.5% agarose. Lymphocytes were treated with UVA (Villingen-Scwennigen, Germany) at wavelength 310–380 nm for 15 min at 37 °C following the comet assay (Anderson et al.^19^). Untreated lymphocytes served as a negative control. Electrophoresis was performed for 30 min at 25 V and the current was adjusted at 300 mA. After electrophoresis, the slides were washed with neutralisation buffer (400 mM Tris–HCl, pH 7.5) three times for 5 min each. For each slide, 100 cells were randomly scored. The parameters used to assess DNA damage were the Olive tail moment (OTM) and % tail DNA.

### Real-time quantitative PCR

Fresh blood from healthy donors and melanoma patients was used for qPCR with prior consent. Lymphocytes from whole blood were isolated using Lymphoprep (3 ml Lymphoprep and 6 ml saline and blood mixture). Cell viability was tested to exclude cytotoxic doses. The viability test included a trypan blue dye exclusion test (10 µl 0.05% trypan blue added to 10 µl cell suspension) and the percentage of live cells was calculated using a haemocytometer under a microscope. The cells were treated with UVA for 15 min by placing them in a casing which consisted of two Waldmann F15/T8-UVA tubes (Villingen-Scwennigen, Germany). A plate with untreated MM and HC cells was used for the purpose of comparison and was not irradiated. RNA samples were isolated using the RNA Isolation Kit (Qiagen) according to the manufacturer’s protocol. The purity and quality of RNA were measured with a NanoDrop™ 1000 spectrophotometer device. cDNA was generated from 1 mg total RNA per sample using the iScript cDNA synthesis kit (Bio-Rad). RT-qPCR was performed using Fast SYBR™ Master Mix (Qiagen) and GAPDH and TP53 primers (Qiagen, UK). GAPDH served as a housekeeping gene for the normalisation of the reaction. The plates were then placed in the Step-One-Plus Real-Time PCR Detection System (Applied Biosystems, Warrington, UK). The conditions were set as follows: denaturation at 95 °C for 10 min, then 40 cycles of denaturation at 95 °C for 15 s, then annealing and extension at 60 °C for 1 min, then at 95 °C for 15 s, 60 °C for 15 s and 95 °C for 15 s for the melting curve.

### Western blotting

Lymphocytes were cultured in 25 ml flasks (Petri dishes) at a concentration of 106 cells/well, incubated overnight and treated with UVA at different intensities for 15 min, with the exception of the negative controls. Cells were then washed twice with cold PBS and lysed by adding 150 μl lysis buffer supplemented with 15 μl fresh protease inhibitor cocktail to the cells. Total protein levels were determined using the BCA Protein Assay Kit (ThermoFisher, UK). Cell lysates were separated using protein electrophoresis and blotted on PVDF membranes (ThermoFisher, UK). The membranes were blocked overnight in 4% bovine serum albumin (BSA) diluted in Tris-buffered saline supplemented with 0.1% Tween 20 at 4 °C (all from Sigma Aldrich, UK). The membranes were then incubated with primary and secondary antibodies (Abcam, UK) (with secondary antibodies at a dilution of 1:1000–10,000) overnight at 4 °C and then for 1 h at room temperature. Then the membranes were washed with wash buffer (1×) three times for 10 min each and visualised using the enhanced luminol-based chemiluminescent (ECL) system. Each experiment was repeated 3 times.

### Genome-wide transcriptome profiling

#### Isolation of total RNA and library preparation

DNase-treated total RNA was extracted from frozen lymphocytes using Maxwell RSC Total Tissue RNA Kit (Promega, Madison, USA). Extracted RNA was quantified by Qubit 4.0 using the RNA HS Assay Kit (ThermoFisher Scientific, Waltham, USA). A maximum of 50 ng of extracted RNA was used to prepare cDNA libraries following the fragmentation of total RNA at 94 °C for 3 min. Whole transcriptome sequencing libraries were prepared using the Takara Smarter Total RNA-Seq Kit V2.5—Pico Input Mammalian (Takara Bio Inc, Kusato, Japan). cDNA libraries were prepared by modified random hexamer priming oligos. During the first PCR amplification, full-length Illumina adapters, including barcodes, were added. The ribosomal cDNA sequences (originating from rRNA) were depleted in the presence of RNase H and the mammalian-specific R-Probes. The remaining fragments were enriched through a second round of PCR amplification, using primers universal to all adapters. The final library contained sequences allowing clustering on any Illumina flow cell. Quality control of libraries was completed on Bioanalyzer (Agilent Technologies, Santa Clara, USA). Each library was quantified to equalise the number of the sequencing input from each sample by the Qubit 4.0 HS Assay Kit (ThermoFisher Scientific, Waltham, USA)^17^

#### NextSeq 500 sequencing, bioinformatic analysis and statistical evaluation

Lymphocytes from MM and HC groups were treated with low dose UVA (0.2 mW/cm^2^) and high dose UVA (1.2 mW/cm^2^) for 15 min and compared to untreated lymphocytes. Treated cells were frozen at − 80 °C for a period of time. All samples were sequenced on a NextSeq (500 Illumina System (Illumina, San Diego, USA)). Paired-end cycle sequencing 2 × 75 was run on the Mid-Output V2.5 Kit, which in total generated a median of 25 million raw paired-end reads/sample. According to TruSeq 96 CD Illumina adapters, Takara indices were used to demultiplex and assign raw sequence reads. Datasets were analysed using tools at Chipster virtual bioinformatics interface at CSC Finland^21^ to process and analyse RNA data for gene expression. Adapters were pre-processed and trimmed. All sequences were quality-checked by FastQC^22^ and paired-end reads were mapped using STAR aligner on *Homo sapiens* genome version release GRCh38.95^23^ Quantitation of sequencing reads in BAM files for each gene was estimated by HTSeq, which resulted in aligned read counts per all sequenced gene transcripts^24^ Differential expression analysis was performed using the DESeq2 Bioconductor package. In brief, normalised control and treatment count tables were merged into one and used as a template for differential expression analysis and to generate fold change values in the log2 scale. Genes with an adjusted cut-off p-value ≤ 0.05 and log2 fold change of + 1 or − 1 were considered significantly expressed. Heatmap and dendrogram of RNA expression profiles were performed using the DESeq 2 package filtering low count values. Volcano plots were drawn using the open-source main Galaxy tools^25^

Results were filtered by descending p-values in DESeq2 analysis following the exclusion of transcripts with low alignment abundancy to evaluate the most significant differentially-expressed genes (DEGs).

### Statistical analysis

Two-way ANOVA was used to compare the mean (± SEM) data of treated MM and HC samples to their respective NCs in the LGS assay and in qPCR. Statistical significance was defined as the following p-values: p < 0.05(*), p < 0.01(**), p < 0.001(***), p < 0.0001 (****), ns (non-significant).

## Results

### LGS assay

**Figure 2:**
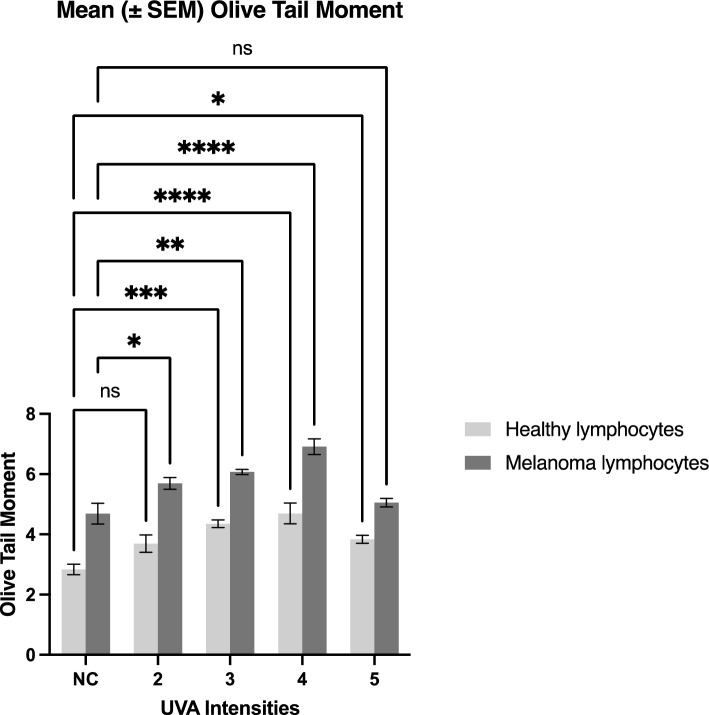
Genotoxicity of UVA on peripheral blood lymphocytes from MM patients and HCs as measured using OTM in the LGS assay. The graph compares the mean (± SEM) OTM values of MM patient samples and HCs, and two-way ANOVA was used to compare the mean (± SEM) OTM of treated MM and HC samples to their respective NCs. Samples were labelled as: NC (negative control, untreated samples), sample 2 (treated with 1.2 mW/cm^2^ UVA), sample 3 (treated with 0.8 mW/cm^2^ UVA), sample 4 (treated with 0.5 mW/cm^2^ UVA, and sample 5 (treated with 0.2 mW/cm^2^ UVA). p < 0.05 (*); p < 0.01 (**); p < 0.001 (***); p < 0.0001 (****), *ns* non-significant).

**Figure 3:**
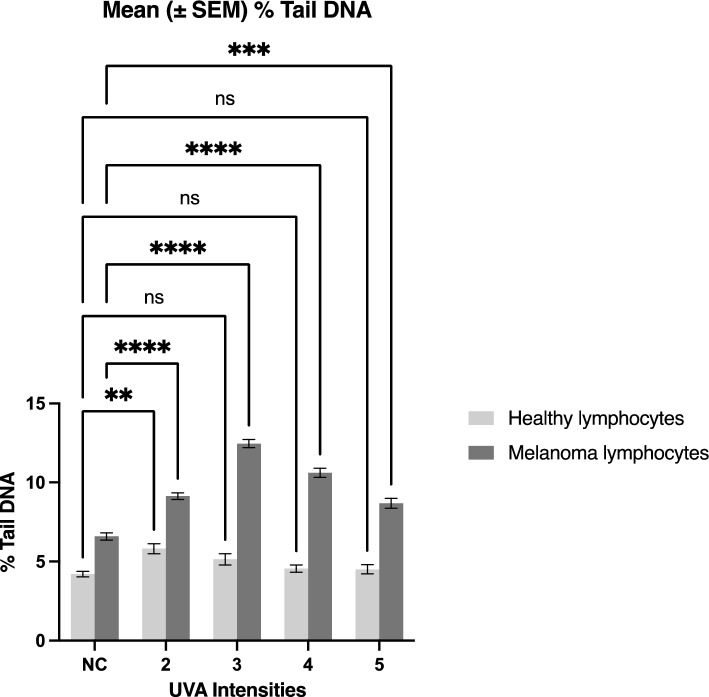
Genotoxicity of UVA on peripheral blood lymphocytes from MM patients and HCs as measured using % tail DNA in the LGS assay. The graph compares the mean (± SEM) % tail DNA values of MM patient samples and HCs, and two-way ANOVA was used to compare the mean % tail DNA (± SEM) of treated MM and HC samples to the respective NCs. Samples were labelled as: NC (negative control, untreated samples), T1 (treated with 1.2 mW/cm^2^ UVA), T2 (treated with 0.8 mW/cm^2^ UVA), T3 (treated with 0.5 mW/cm^2^ UVA, and T4 (treated with 0.2 mW/cm^2^ UVA). p < 0.05 (*); p < 0.01 (**); p < 0.001 (***); p < 0.0001 (****), *ns* non-significant).

The LGS assay was used to determine the mutagenic effect of different UVA intensities on peripheral blood lymphocytes from MM patients and HCs. Our data showed that a UVA intensity of 0.5 mW/cm^2^ caused a significant 1.3-fold increase in DNA damage in lymphocytes from MM patients compared to the negative control (p < 0.0001) and a 1.4-fold increase in DNA damage compared to lymphocytes from HCs, as measured by OTM (Fig. 2). Additionally, UVA intensities of 0.8, 0.5 and 0.2 mW/cm^2^ caused significant 2.4 fold, 2.3 fold and 1.9 fold respective increases in DNA damage in lymphocytes from MM patients compared to HCs, as measured by % tail DNA (p < 0.0001) (Fig. 3).

### Quantitative PCR

**Figure 4:**
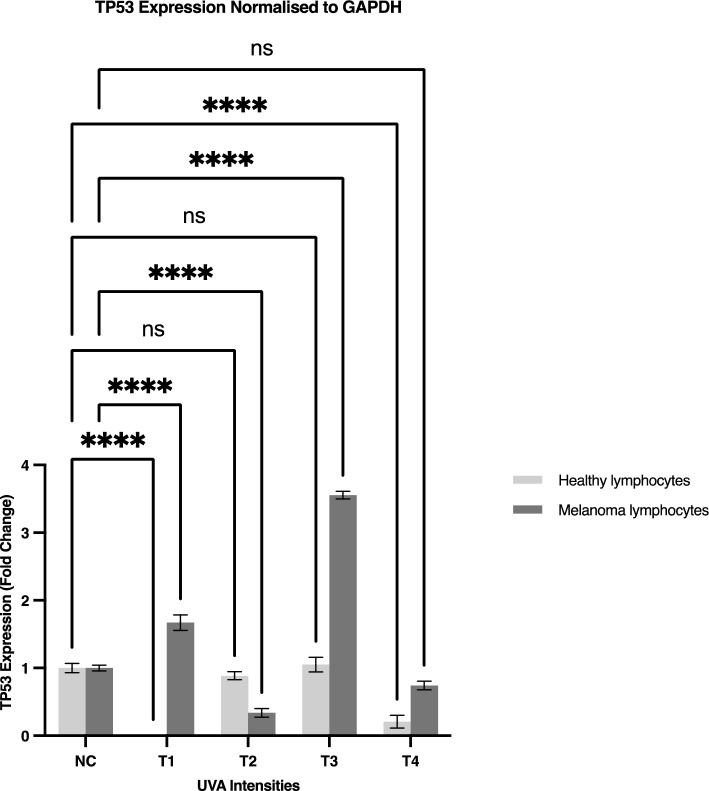
Representative Western blot images showing the expression of wild type and mutant p53 in treated lymphocytes from MM patients and HCs. The treatments are labelled as T1–4, or treatment 1–4 respectively and NC denotes the negative control.

Changes in TP53 expression were observed in qPCR analysis of peripheral blood lymphocytes from both MM patients and HCs following treatment with UVA of various intensities. All expressions were normalised against GAPDH using the ΔΔCt method. The results obtained from each group were compared to their respective negative control. The obtained data demonstrated a 3.4-fold increase in TP53 expression in lymphocytes from MM patients compared to HCs, when irradiated at a UVA intensity of 0.5 mW/cm^2^ (Fig. 4).

### Western blotting

**Figure 5:**
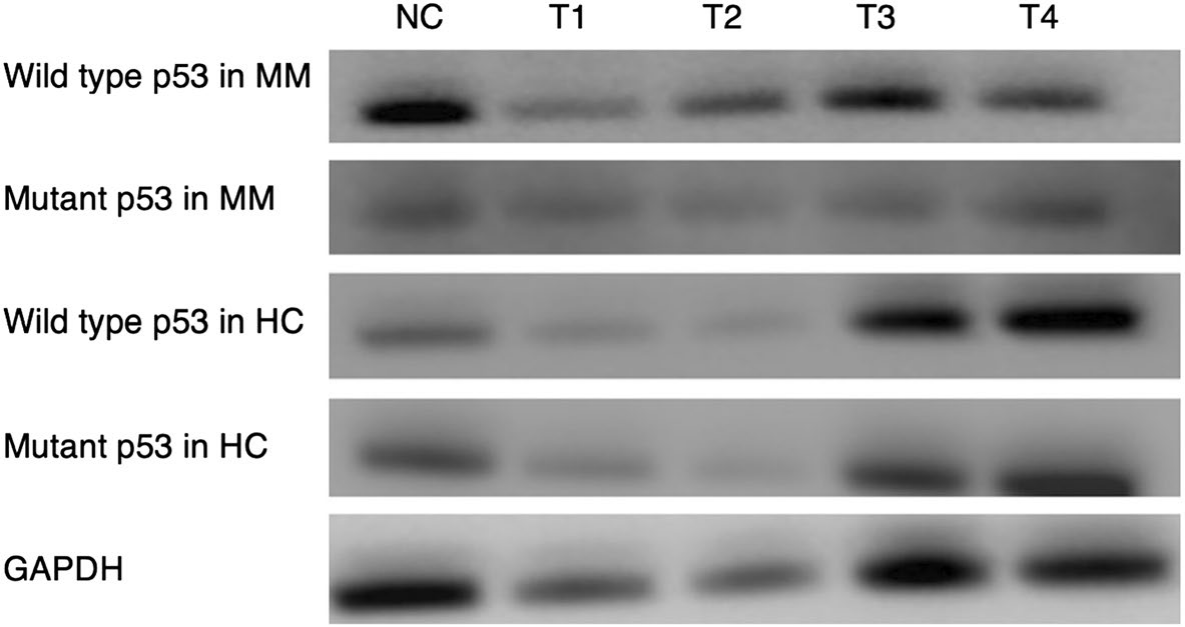
The effect of various UVA intensities on TP53 expression in peripheral blood lymphocytes from MM patients and HCs expression after normalisation with the endogenous housekeeping gene GAPDH in qPCR. Samples were labelled as: NC (negative control, untreated samples), T1 (treated with 1.2 mW/cm^2^ UVA), T2 (treated with 0.8 mW/cm^2^ UVA), T3 (treated with 0.5 mW/cm^2^ UVA), and T4 (treated with 0.2 mW/cm^2^ UVA). Error bars represent the SEM, *p < 0.05. Two-way ANOVA was used to compare the expression of p53 in irradiated lymphocytes of MM patients and HCs to the respective negative controls. p < 0.05 was considered statistically significant. Error bars represent the mean ± SEM. p < 0.0001 (****), *ns* non-significant).

**Figure 6:**
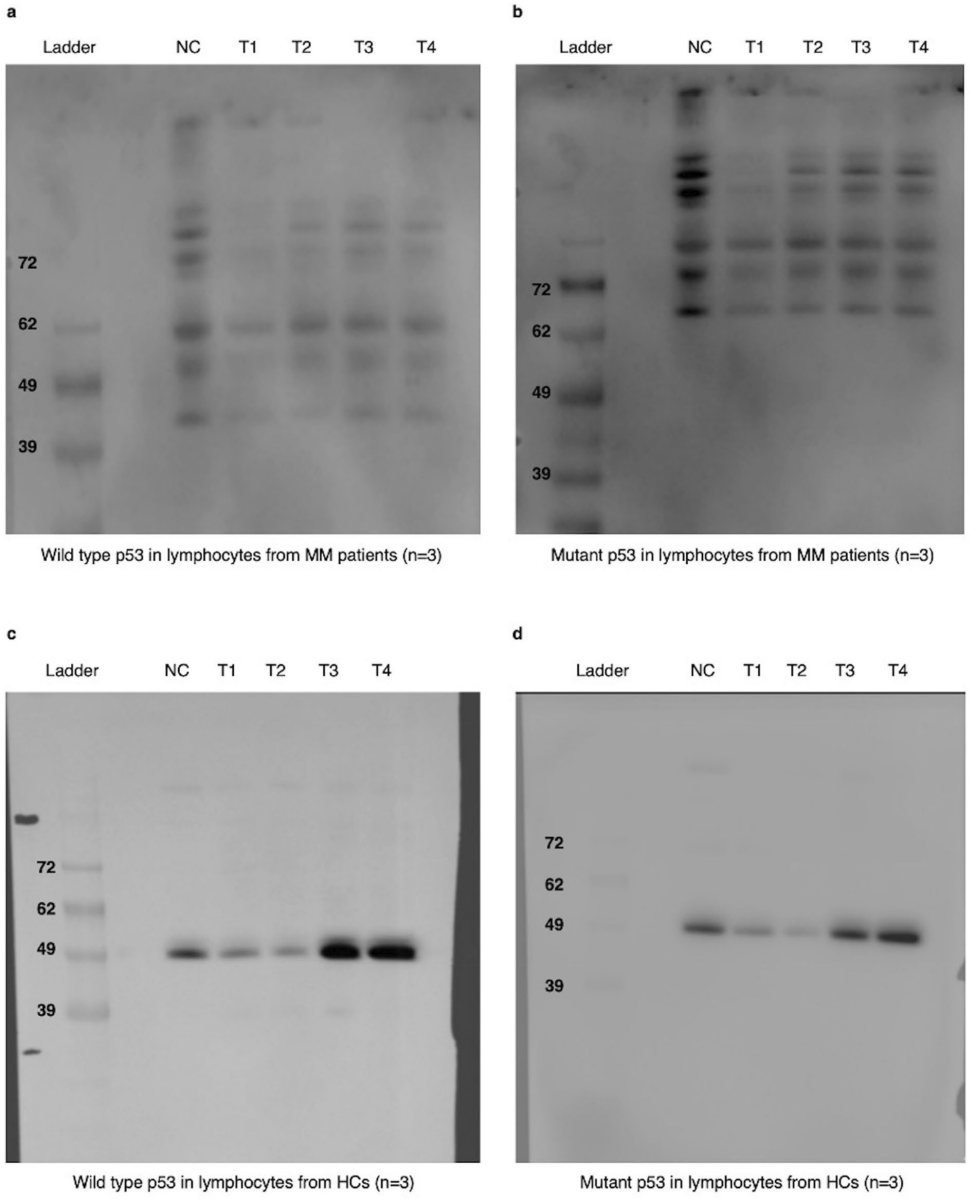
Relative band intensity histograms indicating protein expression in arbitrary values, of wild-type and mutant p53 in lymphocytes from MM patients and HCs in 6 samples (n = 3/group). (**a**) Wildtype and (**b**) mutant p53 expression in treated and nontreated lymphocytes from MM patients. (**c**) Wildtype and (**d**) mutant p53 expression in treated and nontreated lymphocytes from HCs. NC denotes the negative control; T1–T4 (or treatment 1–4) are as follows: T1 (1.2 mW/cm^2^ UVA), T2 (0.8 mW/cm^2^ UVA), T3 (0.5 mW/cm^2^ UVA, and T4 (0.2 mW/cm^2^ UVA). GAPDH protein levels showed equal loading of proteins on the membrane.

We studied the expression levels of p53 wild-type and p53 mutant proteins in lymphocytes from the MM and HC groups before and after treatment with various intensities of UVA. The experiment was repeated three times on 6 different samples (n = 3 in each group). The results showed that following exposure of lymphocytes from both treatment groups to 1.2 mW/cm^2^ UVA, wild-type p53 expression was reduced compared to the respective negative control. However, exposure to 0.8 and 0.5 mW/cm^2^ increased p53 expression in lymphocytes from the MM group compared to HCs (Figs. 5, 6). Additionally, with the same treatments, there was no meaningful difference in the expression of mutant p53 between MM and HC groups (Figs. 5, 6).

### Whole genome transcriptome profiling

**Figure 7:**
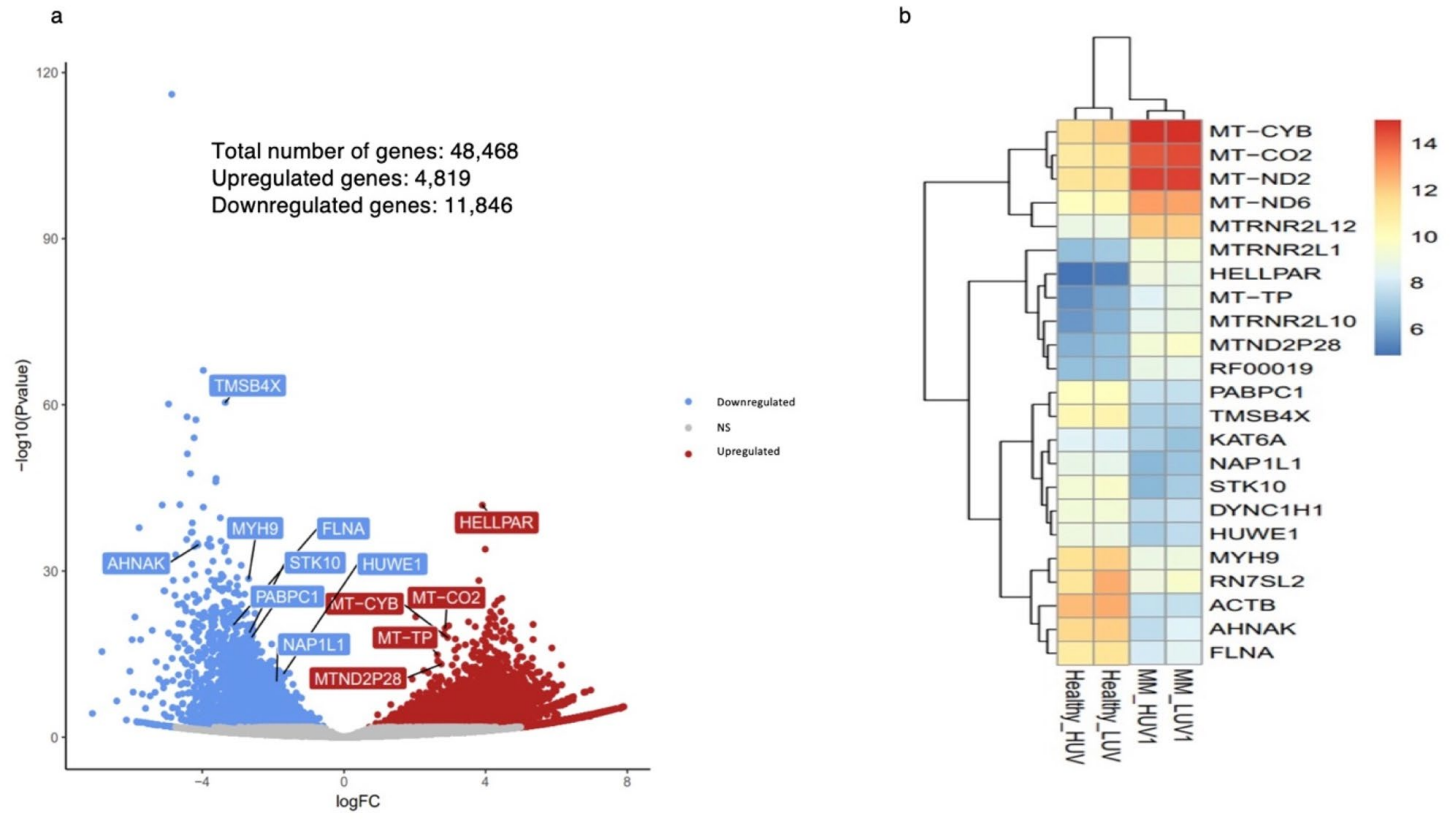
(**a**) Volcano plot of significantly up- and downregulated DEGs. Some of the DEGs are denoted in blue and red boxes. **(b)** Dendrogram and heatmap of 23 DEGs (adjusted p-value < 1e−06). *MM* malignant melanoma, *LUV* low dose (0.2 mW/cm^2^) UVA treatment for 15 min, *HUV* high dose (1.2 mW/cm^2^) UVA treatment for 15 min.

Our results showed significant differential gene expression between UVA-treated lymphocyte samples from two MM patients and two HCs. Analysis of RNA-seq data of samples shows that 11,846 (24%) and 4819 (9.9%) of the total 48,468 counted genes were respectively up- and downregulated (Fig. 7a). Lymphocyte genes in MM samples which were very downregulated compared to those in HC samples included MYH9, RN7SL2, ACTB, AHNAK and FLNA, which play important roles in cell motility, cell structure, cell migration and tumour metastasis (Supplementary Table 1).

Filtering of transcripts with low counts and declining the p-value resulted in 23 DEGs (Fig. 7b; adjusted p-value < 1e−06). Using the Reactome Pathway Database^26^ an over-representation analysis of 23 DEGs was performed, which showed that 5 mitochondrial genes, including MT-CO2, MT-CYB, MT-ND2, MT-ND6 and MTRNR2L12 play an important role in oxidative phosphorylation and respiratory electron transport chain reactions^27,28^ All the highly expressed genes (except one pseudogene) are protein-coding. The proteins they encode and their functions are shown in Table 1.

**Table 1:** Up-regulated and down-regulated genes in MM lymphocytes compared to healthy individuals^27,28^.

	Gene	Protein-coding	Protein	Protein function
Up-regulated	MT-CYB	Yes	Ubiquinol-cytochrome-C reductase complex cytochrome B	Part of the mitochondrial respiratory chain (complex III), mediates electron transfer from ubiquinol to cytochrome c
	MT-CO2	Yes	Cytochrome C oxidase subunit II	Part of respiratory chain (complex IV)
	MT-ND2	Yes	Mitochondrially encoded NADH dehydrogenase 2	Part of mitochondrial respiratory chain (complex I)
	MT-ND6	Yes	Mitochondrially encoded NADH dehydrogenase 6	Involved in mitochondrial electron transport from NADH to ubiquinone and mitochondrial respiratory chain complex I assembly
	MTRNR2L12	Pseudogene	N/A	Plays a role as a neuroprotective and antiapoptotic factor
	MYH9	Yes	Non-muscle myosin heavy polypeptide 9	Cytokinesis, cell motility and maintenance of cell shape
Down-regulated	RN7SL2	Yes	Cytoplasmic ribonucleoprotein complex, signal recognition particle (SRP)	Mediates cotranslational insertion of secretory proteins into the lumen of the endoplasmic reticulum
	ACTB	Yes	Beta-actin	Cell motility, structure, and integrity, and intercellular signaling
	AHNAK	Yes	AHNAK nucleoprotein (desmoyokin)	Play a role in diverse processes such as blood–brain barrier formation, cell structure and migration, cardiac calcium channel regulation, and tumor metastasis
	FLNA	Pseudogene	Filamin A	An actin-binding protein that crosslinks actin filaments and links actin filaments to membrane glycoproteins
	TMSB4X	Yes	Prothymosin beta-4	Plays a role in the regulation of actin polymerisation
	MYH9	Yes	Non-muscle myosin heavy polypeptide 9	Involved in cytokinesis, cell motility and maintenance of cell shape
	PABPC1	Yes	Poly(A)-binding protein, cytoplasmic 2	Promotes ribosome recruitment and translation initiation

A comparison of gene expression between lymphocytes samples from two MM patients and two HCs revealed that out of the 23 genes, 11 and 12 genes, respectively had a higher or lower level of expression in MM patient lymphocytes compared to HCs. Interestingly, 9 out of 23 (~ 40%) of the DEGs that were expressed more highly in MM samples were mitochondrial genes.

DESeq2 expression analysis did not detect any significant differences in gene expression between two untreated MM samples and 4 UVA-treated MM samples, nor was any difference detected between high- and low-intensity UVA-treated samples.

Taken together, it may be inferred that lymphocytes from MM patients and HCs show a different gene expression profile, particularly in mitochondrial genes.

## Discussion

The mutagenic effects of UVA on the skin are well established and are described as one of the major health risk factors which induce melanoma. UVA radiation also causes sunburn, ageing, tanning, and immune suppression. UVA is absorbed by melanin-producing cells in the skin, which promotes oxidative DNA damage^29,30^

Prolonged exposure to known risk factors induces high levels of cellular toxicity in the body, the effects of which may include DNA damage and initiation of the development of different carcinomas. Since peripheral blood lymphocytes circulate freely throughout the body, their exposure to a variety of physical and chemical insults may promote DNA damage and the induction of oxidative stress. Hence, the analysis of peripheral blood lymphocytes can provide insights into the origins of cellular damage. It is well-documented that due to their high availability in blood, lymphocytes serve as ideal surrogates in evaluating the different types of DNA damage that exist in various stages and types of carcinomas^31^

The LGS assay, also known as a modified comet assay, was introduced in 2014 as a simple blood test to detect cancer, based on the enhanced response of lymphocytes from cancer patients to UVA exposure at different intensities compared to healthy individuals^32^

In our study, evaluation of the genotoxic effects of UVA on peripheral blood lymphocytes using the LGS assay revealed significant increases in DNA damage in MM and HC groups compared to that of the negative control when treated using various UVA intensities (p < 0.05). DNA damage induced at different UVA intensities was assessed to determine which intensity caused the maximum genomic damage following exposure, as measured by OTM and % tail DNA (Figs. 2, 3).

Our results are consistent with a previous study which reported an increase in DNA damage in groups of malignant MM patients compared to HCs after treatment with UVA^31^ Furthermore, another published study indicated that significantly high levels of DNA damage in lymphocytes are observed in cancer patients compared to healthy groups; based on these levels of DNA damage, diagnostic tests may be conducted^19,33^

It is well established that p53 is a tumour suppressor protein which inhibits the malignant progression of various tumours in response to cellular oxidative stress^34–36^ One of the vital cellular roles of p53 is the regulation of ROS levels in determining cellular fate. As cells are subjected to different levels of ROS, several studies have indicated the role of the p53 protein in response to cellular stress and in maintaining the homeostasis of ROS levels^36–38^ O’Farrell et al., evaluated the effect of wild-type p53 and mutations in the DNA-binding domain (A138P and R175H) on gene expression, and it was found that < 5% of regulatory abilities remained following P53 mutation. R175H displayed equivalent numbers of wild-type regulatory and novel events, whilst conversely, A138P held greater wild-type regulatory activities^39^

Mutations in, and overexpression of p53 protein have been described in a large proportion of carcinomas^40^ Reports indicate different mutational frequencies of p53 in metastatic melanoma. These studies suggest the importance of p53 stabilisation at the initial stages of cancer. In melanoma, the p53 protein is suggested to exist in the wild-type form. As such, other factors such as DNA damage, oxidative stress, oncogenic and genotoxic events may contribute to its alteration. Several studies have observed the frequency of p53 mutations in melanoma and its association with UV radiation^29,41^

The mentioned hypothesis of the different responses of MM and healthy lymphocytes to UVA exposure was tested and further confirmed using RNAseq data to quantify gene expression. By comparing the gene expression of healthy and MM patient lymphocytes, significant differences were observed between the two groups, especially in mitochondrial genes. Interestingly, some mitochondrial genes (e.g. cytochrome c oxidase) involved in the oxidative phosphorylation pathway were overexpressed in MM patient lymphocytes. Cytochrome c oxidase is a metabolic checkpoint that plays a vital role in controlling cell fate decisions through the activation and proliferation of T-cells. Since oxidative phosphorylation and cytochrome c oxidase activity are increased during T-cell activation^42^ higher expression of cytochrome c oxidase genes in patient lymphocytes may be related to greater activation and proliferation of lymphocytes. This may be seen in patient lymphocytes more than in healthy individuals’ lymphocytes and lead to a noticeable difference in their behaviour (Table 1) and the progression of the disease^41^ The significance of our qPCR findings is that it enables the evaluation of the correlation between UVA and TP53 gene expression. The lymphocytes from MM patients and HCs permitted an overview of the variation existing in normal skin and an insight into the varying sensitivities resulting from exposure to radiation at different intensities in these cells. The qPCR analysis revealed increases in the expression of TP53 in peripheral blood lymphocytes from melanoma patients compared to healthy donors following treatment with 1.2, 0.5 and 0.2 mW/cm^2^ (Fig. 4). These findings are supported by existing literature, which suggests that TP53 is expressed at different levels in non-cancerous naevi compared to MM patients^43^ In addition, another study demonstrated that the upregulation of TP53 could be suggestive of cytotoxicity in the cell, which indicates that TP53 plays an important role in maintaining the integrity and stability of cells^44^

Lymphocytes from MM patients exhibited a greater increase in p53 expression in comparison to lymphocytes from HCs. As such, we note that the observed upregulation in TP53 gene expression may be due to UVA-induced DNA damage, which is associated with the absorption rate of different wavelengths. Our findings are also supported by studies in which elevated levels of TP53 and DNA damage were detected following exposure to sunlight, as compared to the average normal levels of p53 in the epidermis^45–47^.

Genome-wide transcriptome profiling is an advanced method used to measure cell RNA expression^17^ Annotated transcripts were used here to compare differentially expressed genes in lymphocytes from MM patients and a HC group. Non-mapped reads were analysed to study fusion genes or viral transcripts. In the current investigation, the transcriptome sequencing data were used to define novel transcripts, leading to the discovery of neoantigens. The results showed significant and meaningful differences in gene expression in lymphocytes from MM patients compared to HCs following UVA treatment (p-value < 0.05) (Fig. 7). It is the first time that this technique has been applied to the analysis of lymphocytes from cancer patients compared to healthy individuals. Exposure to different UV intensities did not cause a significant difference in the up- or downregulation gene expression in lymphocytes from MM patients or HCs (Fig. 7).

Over-representation analysis on the Reactome Pathway Database^26^ (Fig. 7) displayed 5 mitochondrial genes, MT-CO2, MT-CYB, MT-ND2, MT-ND6 and MT-TP, which play an important role in oxidative phosphorylation and respiratory electron transport chain reactions, processes which are affected in the lymphocytes of MM patients (Fig. 7b).

In Table 1, one of the most indicative upregulated gene sets related to cytochrome c activity. The main role of the cytochrome c protein is to transport electrons in the mitochondrial respiratory chain. However, its accessory mitochondrial function is to induce living cells to apoptosis^48^

## Conclusion

In this investigation, peripheral blood lymphocytes were used as surrogates to detect DNA damage induced by UVA radiation. Our data from the comet assay indicated that UVA radiation caused a significant increase in DNA damage in lymphocytes from MM patients compared to HCs for both OTM and the percentage tail of the DNA. The results from real-time quantitative PCR revealed a significant increase in p53 expression in lymphocytes from MM patients compared to HC samples. Additionally, genome-wide transcriptome analysis confirmed a genome difference between lymphocytes from MM patients compared to HCs following UVA treatment. This study shows that different sensitivity levels exist in MM patients and HCs after exposure to an external genotoxic insult, such as UV radiation. The results in all three of our assays revealed genomic changes after UVA exposure. However, the DNA damage found in lymphocytes from MM patients exposed to ultraviolet radiation was much greater than the untreated samples from the same individuals.

Our findings indicate that even the lowest intensity of UVA exposure has potentially harmful effects on MM patient lymphocytes. Irreversible changes in the DNA may lead to MM and other types of skin cancer. Studies suggest that peripheral blood lymphocytes—due to their availability in the circulatory system—may carry greater intrinsic damage from genotoxic insults and show greater responsivity compared to other cells in the body. Based on our results, this feature of peripheral blood lymphocytes of exhibiting different sensitivity levels in each of HCs, precancerous and cancerous patients, could be used as a biomarker in the future. Using this sensitivity for the screening and diagnosing precancerous and/or early stages of cancers may provide a window of opportunity for timely intervention to prevent progression from precancerous to cancerous or further to metastatic stages.

### Supplementary Information

Supplementary Table 1.

Supplementary Information.

## Data Availability

All the raw data related to this article is available to the editors upon request. The datasets used and/or analysed in this study are available from the corresponding author upon reasonable request.
