# Supplementary material for: Comparing P53 expression and genome-wide transcriptome profiling to Comet assay in lymphocytes from melanoma patients and healthy controls
Source: Sci Rep. 2023 Nov 1;13:18858. doi: 10.1038/s41598-023-44965-z (PMC10620420; doi:10.1038/s41598-023-44965-z)
Supplement: Supplementary file 1 — Supplementary Table 1. [file 41598_2023_44965_MOESM1_ESM.docx]

|  | **Gene name** | **Protein coding** | **Protein name** | **Function protein** |
| --- | --- | --- | --- | --- |
| Up-regulated | MT-CYB | yes | Ubiquinol-Cytochrome-C Reductase Complex Cytochrome B | complex III , part of the mitochondrial respiratory chain, mediates electron transfer from ubiquinol to cytochrome c. |
|  | MT-CO2 | yes | Cytochrome C Oxidase Subunit II | Part of respiratory chain complex IV. |
|  | MT-ND2 | yes | Mitochondrially Encoded NADH Dehydrogenase 2 | Part of mitochondrial respiratory chain complex I |
|  | MT-ND6 | yes | Mitochondrially Encoded NADH Dehydrogenase 6 | Involved in mitochondrial electron transport, NADH to ubiquinone and mitochondrial respiratory chain complex I assembly. |
|  | MTRNR2L12 | Pseudogene | - | Plays a role as a neuroprotective and antiapoptotic factor |
|  | MYH9 | yes | Non-Muscle Myosin Heavy Polypeptide 9 | cytokinesis, cell motility and maintenance of cell shape |
| Down-regulated | RN7SL2 | yes | cytoplasmic ribonucleoprotein complex , signal recognition particle (SRP) | Mediates cotranslational insertion of secretory proteins into the lumen of the endoplasmic reticulum. |
|  | ACTB | yes | Beta-Actin | cell motility, structure, integrity, and intercellular signaling |
|  | AHNAK | yes | AHNAK Nucleoprotein (Desmoyokin) | Play a role in such diverse processes as blood-brain barrier formation, cell structure and migration, cardiac calcium channel regulation, and tumour metastasis. |
|  | FLNA | Pseudogene | Filamin A | An actin-binding protein that crosslinks actin filaments and links actin filaments to membrane glycoproteins. |
|  | TMSB4X | yes | Prothymosin Beta-4 | plays a role in regulation of actin polymerization |
|  | MYH9 | yes | Non-Muscle Myosin Heavy Polypeptide 9 | involved in cytokinesis, cell motility and maintenance of cell shape. |
|  | PABPC1 | yes | Poly(A)-Binding Protein, Cytoplasmic 2 | promotes ribosome recruitment and translation initiation; |

***Supplementary Table 1.*** *Up-regulated and down-regulated genes in MM lymphocytes compared to healthy individuals (Stelzer et al., 2016, Barshir et al., 2021)*
