# Supplementary figures and images for: Comparing P53 expression and genome-wide transcriptome profiling to Comet assay in lymphocytes from melanoma patients and healthy controls
Source: Sci Rep. 2023 Nov 1;13:18858. doi: 10.1038/s41598-023-44965-z (PMC10620420; doi:10.1038/s41598-023-44965-z)

**Supplementary:**

**Wild P53 in MM**


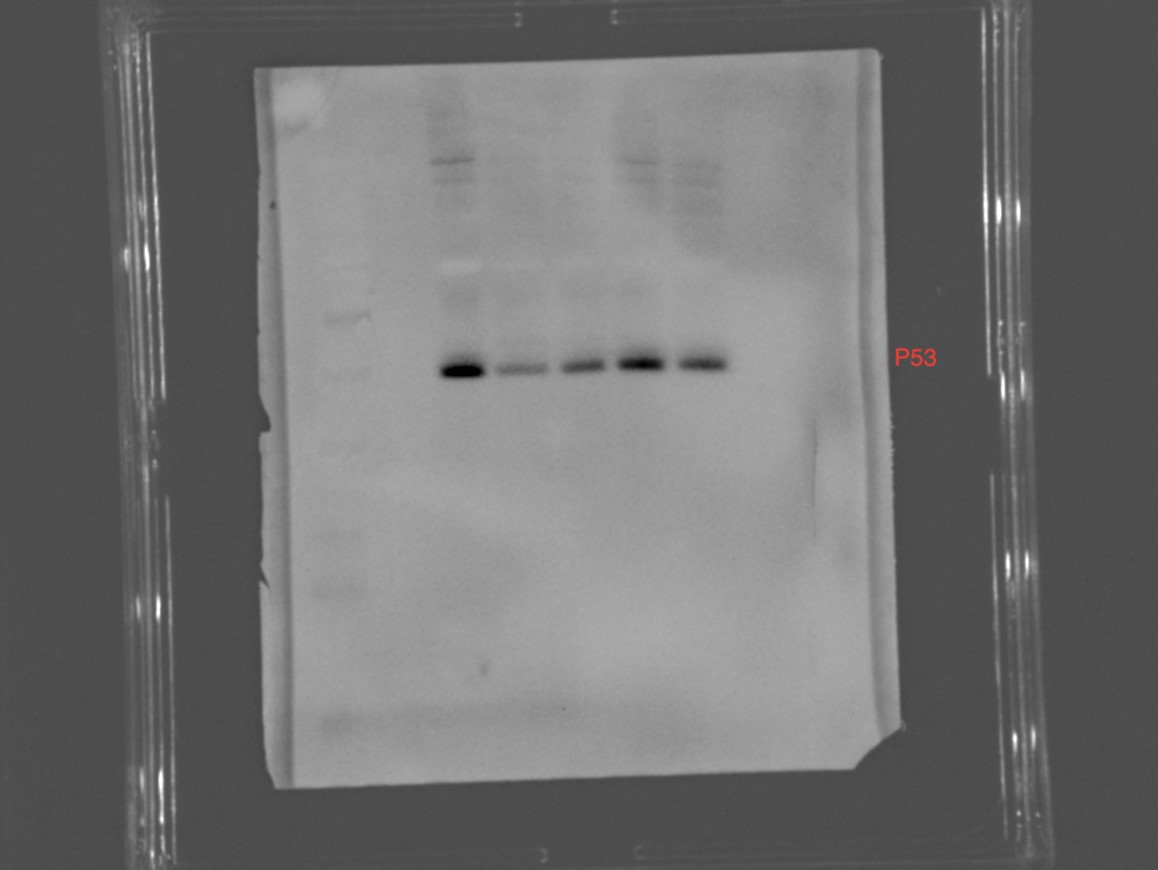


Mutant P53 in MM


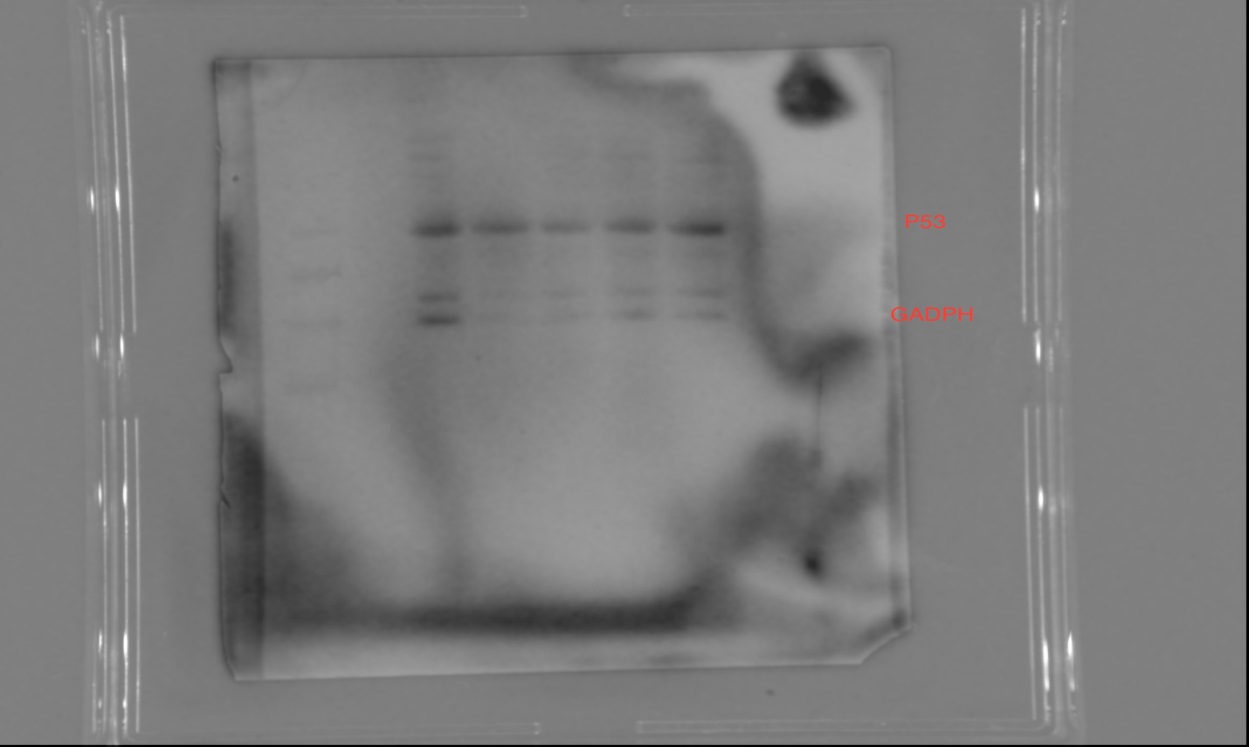


Wild P53 in healthy


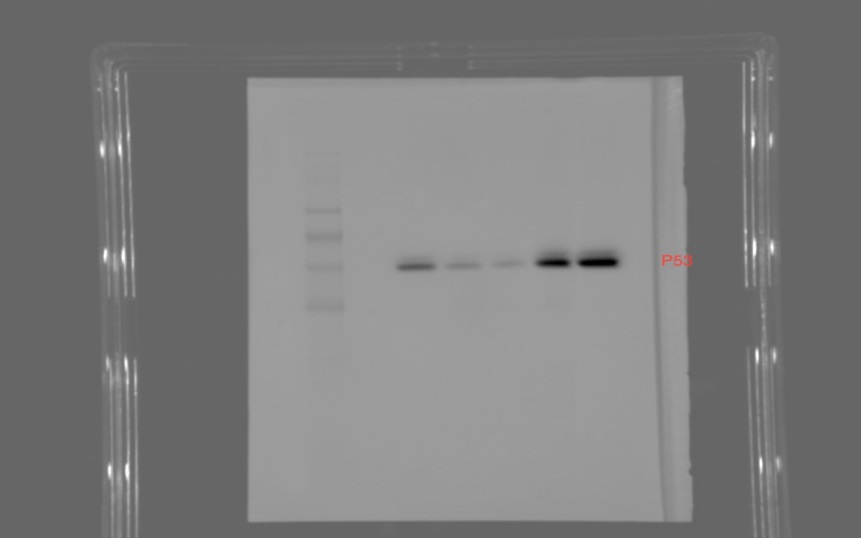


**Mutant P53 Healthy**


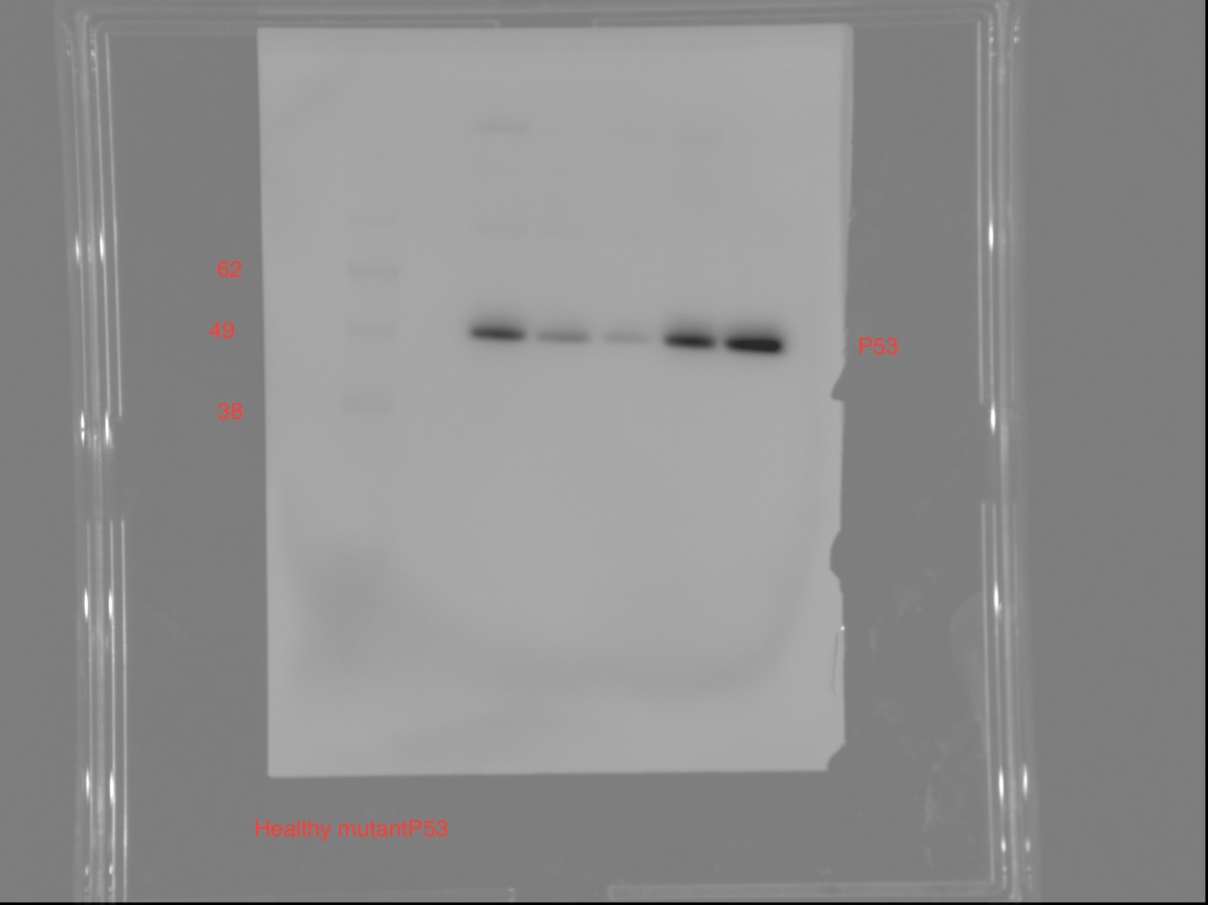


**GAPDH**


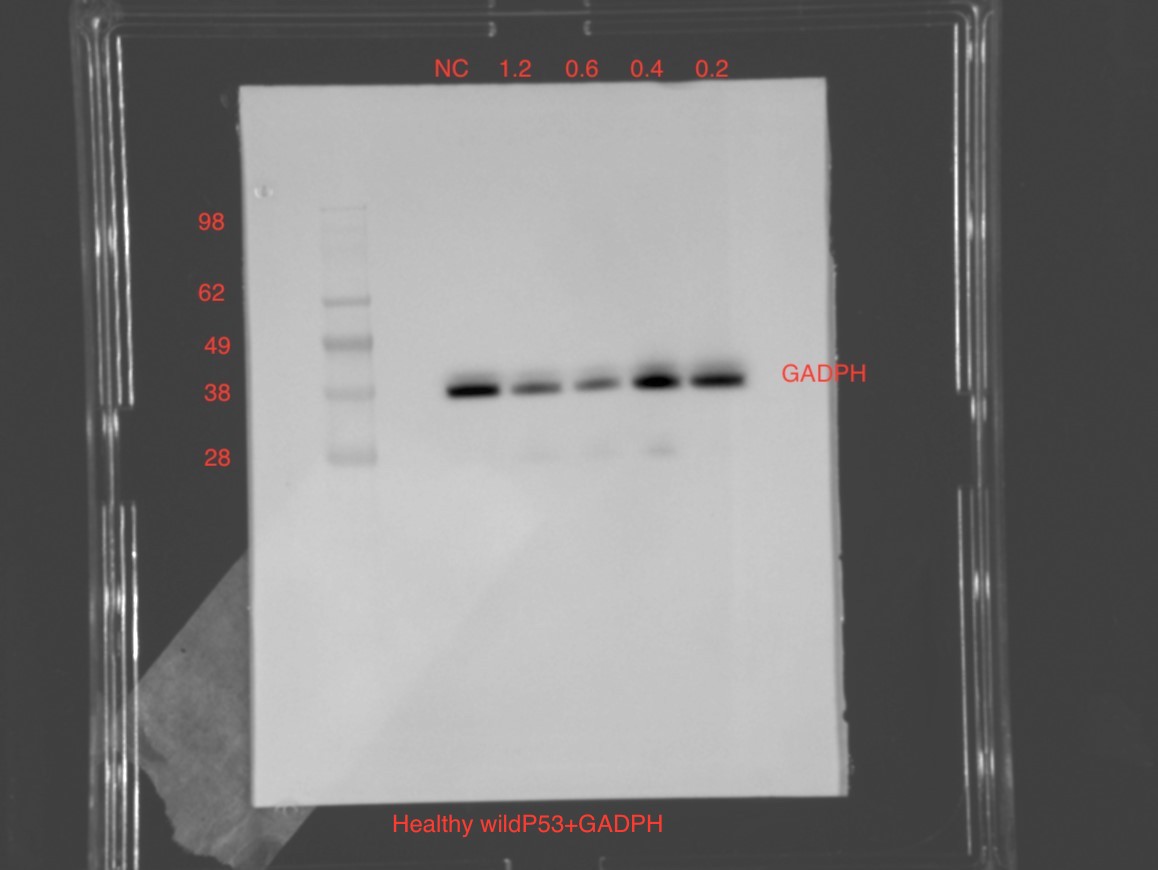

Supplement: Supplementary file 2 — Supplementary Information. [file 41598_2023_44965_MOESM2_ESM.docx]
